# A tryptophan metabolite prevents depletion of circulating endothelial progenitor cells in systemic low-grade inflammation

**DOI:** 10.3389/fimmu.2023.964660

**Published:** 2023-04-04

**Authors:** Massimo R. Mannarino, Vanessa Bianconi, Giulia Scalisi, Luca Franceschini, Giorgia Manni, Alessia Cucci, Francesco Bagaglia, Giulia Mencarelli, Francesco Giglioni, Doriana Ricciuti, Filippo Figorilli, Benedetta Pieroni, Elena Cosentini, Eleonora Padiglioni, Cecilia Colangelo, Dietmar Fuchs, Paolo Puccetti, Antonia Follenzi, Matteo Pirro, Marco Gargaro, Francesca Fallarino

**Affiliations:** ^1^ Department of Medicine and Surgery, University of Perugia, Perugia, Italy; ^2^ Department of Health Sciences, School of Medicine, University of Piemonte Orientale, Novara, Italy; ^3^ Division of Biological Chemistry, Biocenter, Innsbruck Medical University, Innsbruck, Austria

**Keywords:** EPC — endothelial progenitor cells, kynurenine (KYN), IDO1 enzyme, low-grade inflammation, IL-6 (interleukin 6), hs-CRP (high sensitivity C-reactive protein)

## Abstract

**Background:**

Chronic systemic inflammation reduces the bioavailability of circulating endothelial progenitor cells (EPCs). Indoleamine 2,3-dioxygenase 1 (IDO1), a key enzyme of immune tolerance catalyzing the initial step of tryptophan degradation along the so-called l-kynurenine (l-kyn) pathway, that is induced by inflammatory stimuli and exerts anti-inflammatory effects. A specific relationship between IDO1 activity and circulating EPC numbers has not yet been investigated.

**Methods:**

In this study, circulating EPCs were examined in mice treated with low doses of lipopolysaccharide (LPS) to mimic low-grade inflammation. Moreover, the association between IDO1 activity and circulating EPCs was studied in a cohort of 277 patients with variable systemic low-grade inflammation.

**Results:**

Repeated low doses of LPS caused a decrease in circulating EPCs and l-kyn supplementation, mimicking IDO1 activation, significantly increased EPC numbers under homeostatic conditions preventing EPC decline in low-grade endotoxemia. Accordingly, in patients with variable systemic low-grade inflammation, there was a significant interaction between IDO1 activity and high-sensitivity C-reactive protein (hs-CRP) in predicting circulating EPCs, with high hs-CRP associated with significantly lower EPCs at low IDO1 activity but not at high IDO1 activity.

**Interpretation:**

Overall, these findings demonstrate that systemic low-grade inflammation reduces circulating EPCs. However, high IDO1 activity and l-kyn supplementation limit circulating EPC loss in low-grade inflammation.

## Introduction

Endothelial progenitor cells (EPCs) derived from myeloid pluripotent stem cells play a significant role in maintaining endothelial homeostasis through their regenerative and reparative functions (1). The availability of circulating EPCs is significantly reduced by chronic systemic inflammation, either clinically manifest or subclinical (2, 3). Indeed, the chronic exposure of bone marrow to pro-inflammatory cytokines may impair the proliferation, mobilization, and survival of EPCs (2, 3). Accordingly, in different chronic inflammatory diseases, including rheumatoid arthritis, systemic lupus erythematosus, and systemic sclerosis, reduced peripheral levels of EPCs have been reported (3, 4). Also, in patients exposed to different cardiovascular risk factors and/or with stable atherosclerotic cardiovascular disease, which may drive a status of chronic subclinical inflammation, decreased circulating EPC counts have been described and a negative correlation has been reported between circulating EPCs and hs-CRP, one of the best surrogate parameters of systemic inflammation (5–8). Conversely, different drugs with anti-inflammatory properties have been shown to improve the availability of circulating EPCs in different chronic inflammatory conditions (4, 9, 10). Noteworthy, among pro-inflammatory cytokines, tumor necrosis factor (TNF)-α and interleukin (IL)-6 are considered key players of EPC depletion in chronic inflammation (4). Indeed, previous experimental studies have shown that the chronic exposure of EPCs to TNF-α and IL-6 can induce a significant reduction of cell proliferation and migration, as well as increased cell apoptosis rates (4, 11, 12). In addition, there is evidence that EPC exposure to lipopolysaccharide (LPS), a well-known mediator of chronic subclinical inflammation (12, 13), may induce an increased expression of pro-apoptotic factors leading to reduced EPC survival (14). Indoleamine 2,3-dioxygenase 1 (IDO1) is an inducible heme protein that catalyzes the initial step of tryptophan (Trp) degradation along the so-called l-kynurenine (l-kyn) pathway (15). IDO1 has both enzymatic and non-enzymatic regulatory functions (16), and it is a pivotal component of a complex, pleiotropic system that allows for the long-term control of immune homeostasis (17, 18). Tryptophan degradation by IDO1 yields l-kyn (19, 20), that regulates immune homeostasis mainly by acting as a ligand for aryl hydrocarbon receptor (AhR). Currently, despite some inconsistency, there is a large body of evidence supporting the notion that IDO1 enzymatic activity may result in anti-inflammatory effects in different inflammatory conditions (21–25). Accordingly, it would be of interest to assess whether IDO1 activity can influence the negative association between chronic systemic inflammation and EPC bioavailability. In this regard, it is plausible that IDO1 enzymatic activity, due to its ability to attenuate inflammatory responses, might interfere with chronic systemic inflammation-induced EPC loss. This study was aimed 1) at comparing circulating EPCs levels between mice with low dose endotoxemia and mice with low dose endotoxemia supplemented with l-kyn, and 2) at assessing the association between hs-CRP and circulating EPCs according to IDO1 activity in a human cohort of patients exposed to variable systemic low-grade inflammation.

## Materials and methods

### Mice

C57BL/6 mice, referred to as WT mice, were purchased from Charles River Breeding laboratories and *Ido1^–/–^* mice were obtained from The Jackson Laboratory. All mice were male and 8-10 weeks old on commencing the experiments. They were all housed and fed under specific pathogen-free conditions. All *in vivo* studies were approved by the Ethical Committee of the University of Perugia and done in compliance with National Animal Care and Use Committee guidelines.

For induction of systemic low-grade inflammation, mice were given daily intravenous injections with a low dose of LPS (Sigma #L2880) (approximately 1µg/kg/mouse; ˜20 ng/mouse) dissolved in 200 µL of endotoxin-free phosphate buffered saline (PBS) for 3 consecutive weeks. For l-kyn (Sigma #K8625) supplementation, mice received l-kyn (5 mg/mouse) by oral gavage 3 times weekly for 3 consecutive weeks, either alone or in combination with LPS. For control treatment, mice were given daily intravenous injections of PBS for 3 consecutive weeks. Blood and spleens samples of WT mice were collected on day 0, day 15 and day 22.

For IDO1 and TDO2 inhibition *in vivo*, Epacadostat (5 mg/kg per mouse; Selleckchem) and 680C91 (5 mg/kg; R&D Systems) were administered respectively by oral gavage for three consecutive days. Vehicle-treated groups were used as controls. Blood samples were collected on day 4.

### Cytokines’ analysis

IL-6 (#88-7064-88, Thermo Fisher Scientific) and TNF-α (#88-7324-88, Thermo Fisher Scientific) were measured by specific ELISA kits according to the manufacturer’s recommendation. Briefly, specific anti-cytokine antibodies were coated in Nunc-Immuno 96 MicroWell solid plates (#422404, Thermo Fisher Scientific). Standard curve and samples were incubated 2 hours in coated plates. Subsequently, a detection antibody (biotinylated antibody) was added to bind the immobilized antigen captured during the first incubation. A streptavidin-HPR antibody was added. Luminescence was read at 450 nm (subtracting the value of 570 nm as non-specific plate absorbance) on Tecan microplate reader Spark (26). The detection limits of the assays were 4 pg/ml for IL-6 and 8 pg/ml for TNF-α. Multiplex ELISA was performed by using a 36-plex immunoassay (Cytokine & Chemokine Convenience 36-Plex Mouse ProcartaPlex™ Panel 1A, # EPXR360-26092-901) and a MAGPIX system (Luminex Corporation).

### Staining and quantification of murine circulating EPCs

Murine circulating EPCs were determined by flow cytometry (**Supplementary Figure 1**), Briefly, murine peripheral blood mononuclear cells (PBMCs) were isolated using Ficoll-Paque Plus (#17-1400-02, GE Healthcare) gradient centrifugation from whole blood samples diluted 1:1 with buffered saline (Lymphoprep, Axis-Shield PoC AS, Oslo, Norway). Subsequently, PBMCs were immunolabeled by incubating cell suspensions with fluoresce-conjugated antibodies against murine kinase insert domain receptor (KDR) (R&D Systems, Minneapolis, MN, Clone # 522302), murine stem cell antigen SCA1 (Beckman Coulter, Inc. Fullerton, CA, Clone # 104D2D1) and murine tyrosine-protein kinase KIT (CD117) (Biolegend, Sand Diego, CA, Clone# D7); isotype-matched antibodies served as controls. Quantitative analysis was performed on a Coulter Epics XL (Beckman Coulter) measuring 1,000,000 cells per sample, after exclusion of nonviable mononuclear cells by staining with propidium iodide at a final concentration of 3 mg/mL. Cells were identified following the gating strategy reported in **Supplementary Figure 1**. The number of murine EPCs was calculated by multiplying the frequency of CD117+SCA1+KDR+ events in the leukocyte gate by the total leukocyte count (the CD45+) according to technique previously used by other investigators and us (27). For each mouse the number of EPCs in two separate blood samples was highly reproducible (r=0.90; p<0.001).

### *In vitro* tubulogenic assay

Forty-eight-well tissue culture plates were coated with 150 µl Geltrex™ LDEV-Free Reduced Growth Factor Basement Membrane Matrix (Gibco) per well and allowed to solidify at 37°C for 30 minutes. 2x104 BOECs (or ECFCs) (28) were resuspended in MCDB131 medium (Gibco) supplemented with 1% FBS and placed on top of the matrix. Cells were treated with 50ng/ml of TNF-α in presence or absence of 100µM L-Kynurenine for five days. Vehicle was used as control. Plates were incubated at 37°C, 5% CO2 and analyzed after overnight incubation. Images were acquired under inverted microscope Leica ICC50. ImageJ Angiogenesis software was used for the quantification of number of nodes, junctions, branches, and total length.

### RNA extraction, cDNA synthesis and real-time PCR

Total splenocytes were lysed in RLT buffer (#79216, Qiagen) and RNA was isolated using RNeasy Mini Kit (#74104, Qiagen). cDNA was transcribed using Quantitect Reverse transcription kit (#205313, Qiagen) according to manufacturer’s protocol. *Ido1* and *Ido2* gene expression was measured using iTaq Supermix (#1725124, Bio-rad) using primer the following primers: *Gapdh* 5’ -CTG CCC AGA ACA TCA TCC CT -3’; 5’ - ACT TGG CAG GTT TCT CCA GG -3’; *Ido1* 5’ - CGA TGT TCG AAA GGT GCT GC-3’; 5’ - GCA GGA GAA GCT GCG ATT TC-3’; *Ido2* 5’- CTC AGA CTT CCT CAC TTA ATC G -3’; 5’- GCT CAC GGT AAC TCT -3’. Data were calculated as the ratio to *Gapdh* expression by relative quantification method (ΔΔct; means ± SD of triplicate determination) and data are presented as normalized transcript expression in the samples relative to normalized transcript expression in control cultures (in which fold change = 1).

### Western blotting

Samples were collected, washed in ice-cold 1 X PBS and subsequently resuspended in Lysis buffer (50 mM Tris HCl pH 8, 150 mM NaCl, 1% NP-40, 0.5% sodium deoxycholate, 0.1% SDS) supplemented with protease inhibitors, Aprotin (#A1153, Sigma-Aldrich, 2 ug/ml), Leupeptin (#L9783, Sigma-Aldrich, 10 ug/ml), Pepstatin A (#P5318, Sigma-Aldrich, 1 ug/ml), PMSF (#P7625, Sigma-Aldrich, 1 mM), Sodium Fluoride (#215309 Sigma-Aldrich, 10 mM), Sodium Orthovanadate (#450243, Sigma-Aldrich, 1 mM) and EDTA 5 mM. Cells were lysed by incubation for 30 minutes on ice followed by vortexing each 5 minutes, then centrifugated at 12000 r.p.m. for 30 minutes at 4°C. Protein lysates were quantified using Bradford protein assay (#500-0006, BioRad). 13 μg of protein lysates were boiled at 95°C for 5 minutes. SDS-PAGE was performed using 10% acrylamide gel. Western blotting was performed by transferring proteins onto a nitrocellulose membrane (#1704159, BioRad). Membranes were blocked in 5% non-fat dry milk (#170-6404, BioRad) in 0.1% Tween 20 TBS (TBST) 1 hour at room temperature. Blocked membranes were probed with primary antibodies overnight at 4°C. After washing, membranes were subsequently incubated with secondary antibodies for 1 hour at room temperature in 5% non-fat dry milk in TBST. Murine IDO1, IDO2 and TDO2 were measured using respectively a rabbit anti-murine IDO1 monoclonal antibody (24), a rabbit anti-murine IDO2 and a rabbit anti-murine TDO2 polyclonal antibodies raised in our laboratory. Anti-rabbit (#31460, 1:10000 v/v) secondary horseradish peroxidase-linked antibodies were purchased from Thermo Fisher Scientific Laboratories. Membranes were developed using Clarity Western ECL Blotting Substrates (#170-5061, BioRad) and Clarity Max Western ECL Substrate (#170-5062, BioRad). β-tubulin was used as loading control.

### l-kynurenine determination

Plasma IDO1 enzymatic activity was measured in terms of ability to metabolize Trp to l-kyn, whose concentrations were measured by high-performance liquid chromatography (high-performance liquid chromatography) (29). Briefly, human and murine plasma samples were collected and kept frozen at -20°C until analysis. l-kyn concentrations were measured by HPLC. The detection limit of the assay was 0.05 μM. the l-kyn/Trp ratio, referred to as IDO1 activity, was calculated dividing l-kyn (μmol/L) by Trp concentrations (mmol/L) concentrations (30).

### Patient recruitment

Two-hundred and seventy-seven patients attending the Unit of Internal Medicine, Angiology and Arteriosclerosis Diseases (Perugia, Italy) for cardiovascular risk assessment were recruited. Patients with any one of the following conditions were excluded: clinically manifest cardiovascular disease, renal impairment, liver failure, clinical/laboratory evidence of concomitant inflammatory diseases, and/or ongoing anti-inflammatory therapies. Informed consent was obtained before enrollment, according to a protocol approved by the Ethical Committee of the University of Perugia, in compliance with the Declaration of Helsinki. All determinations were made at a room temperature between 21°C and 23°C, following overnight fast (12-13 hours). For each patient height, weight, and waist circumference measurements were taken to the nearest 0.1 cm, 0.1 cm, and 0.1 Kg respectively. Body mass index (BMI) was calculated as weight (Kg) divided by height squared (m^2^). Brachial blood pressure was measured using a sphygmomanometer, after patients had sat ten minutes or longer; the average of three sequential measurements was recorded (31, 32). Venous blood samples were collected using routine techniques. Total cholesterol, triglycerides, high-density lipoprotein (HDL) cholesterol and glucose were determined by an enzymatic colorimetric method (Autoanalyzer KONE-PRO; DASIT S.p.A, Cornaredo, Milano, Italy); low-density lipoprotein (LDL) cholesterol was calculated by the Friedewald equation. The estimated glomerular filtration rate (eGFR) was calculated through the Chronic Kidney Disease Epidemiology Collaboration (CKD-EPI) equation. Plasma hsCRP levels were measured using nephelometry (BN100; Siemens Dade Behring, Siemens S.p.A., Milan, Italy).

### Quantification of human circulating EPCs

Human circulating EPCs were determined by flow cytometry (**Supplementary Figure 2**). Briefly, human PBMCs were isolated using Ficoll-Paque Plus (#17-1400-02, GE Healthcare) gradient centrifugation from whole blood samples diluted 1:1 with buffered saline (Lymphoprep, Axis-Shield PoC AS, Oslo, Norway). Subsequently, PBMCs (approximately 3106 cells) were immunolabeled by incubating the cell suspensions with fluorescence-conjugated antibodies against human KDR (R&D Systems, Minneapolis, MN, Clone # 89106) and human CD34 (Beckman Coulter, Inc. Fullerton, CA, Clone # 581); isotype-matched antibodies served as controls. Quantitative analysis was performed on a Coulter Epics XL (Beckman Coulter) measuring 1,000,000 cells per sample, after exclusion of nonviable mononuclear cells by staining with propidium iodide at a final concentration of 3 mg/mL. The number of human EPCs was calculated by multiplying the frequency of CD34^+^/KDR^+^ events in the leukocyte gate by the total leukocyte counts (the CD45+) (31, 33). For each subject the EPC counts in two separate blood samples were highly reproducible (r=0.92; p<0.001).

### Statistical analysis

#### Animal study

The SPSS statistical package version 24.0 (SPSS Inc, Chicago, USA) and the GraphPad Prism version 9.3.1. (GraphPad Software, San Diego, USA) were used for statistical analyses. All n values were computed by power analysis to yield a power of at least 80% with an α-level of 0.05. All values from *in vitro* and *in vivo* determinations were collected as means ± SD of at least two independent experiments. Logarithm (lg) transformation was performed when appropriate for non-parametric variables. The independent samples *t* test and the paired samples *t* test were used to compare variables between two groups, as appropriate. The one-way ANOVA with the Bonferroni *post hoc* analysis was used to compare variables between multiple groups. The General Linear Model (GLM) repeated measures test with the Bonferroni *post hoc* analysis was performed to compare variables between multiple groups according to different treatments. Statistical significance was assumed if a null hypothesis could be rejected at p=0.05. All statistical tests and n values are included in the Figure Legends, with all data being shown as mean ± SD and error bars indicating SD. In all cases, **** P <0.0001, *** P <0.001, ** P <0.01, * P <0.05, and ns = not statistically significant (i.e., p ≥0.05).

#### Human study

The SPSS statistical package version 24.0 (SPSS Inc, Chicago, USA) was used for statistical analyses. Continuous variables were summarized as the mean ± SD or as the median (25^th^-75^th^ percentiles). Categorical variables were summarized as numbers and percentages. Lg-transformation was performed when appropriate for non-parametric variables. High hs-CRP was defined by a serum level >3 mg/L according to evidence reporting this cut-off value as that with the best discrimination power between high and low/intermediate cardiovascular risk among patients exposed to cardiovascular risk factors (34), whereas high IDO1 activity by an absolute l-kyn/Trp level ≥40 (i.e., the median value in the study population). The independent sample *t* test, the Mann Whitney U test and the χ2 test were used to compare variables between the high versus low IDO1 activity subgroups, as appropriate. Correlation analyses were performed using the Pearson’s coefficient of correlation. The GLM univariate analysis was used to evaluate the p values of the interaction terms between high versus low IDO1 activity and high versus low hs-CRP in the prediction of circulating EPCs. Statistical significance was assumed if a null hypothesis could be rejected at p=0.05.

## Results

### Repetitive low doses of LPS induce systemic low-grade inflammation reducing circulating EPCs

LPS is the major physiologically active substance derived from bacteria, especially the Gram-negative bacteria frequently seen as part of the gut flora. Low amounts of blood circulating endotoxin, most likely derived from mucosal leakages, play an important part in low-grade chronic inflammatory diseases (12).

**Figure 1:**
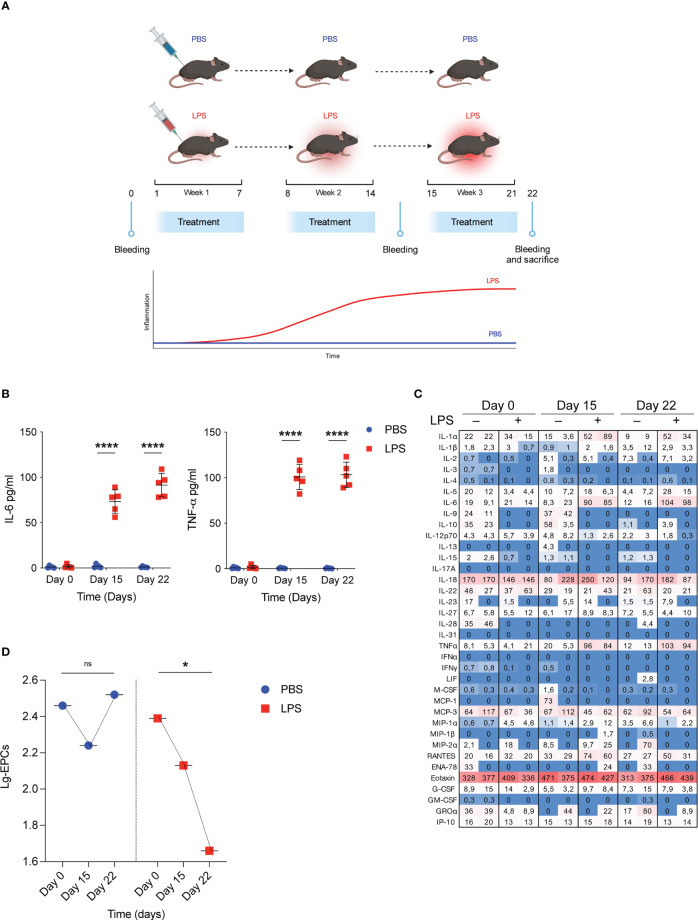
Low grade of inflammation reduces circulating EPCs. **(A)** WT mice were treated with low doses of LPS for 3 weeks. On day 0, day 15 and day 22 bleeding was performed for the determination of circulating EPCs, serum cytokines, and serum IDO1 activity; subsequently, mice were sacrificed for the determination of Ido1 mRNA and IDO1 protein expression in splenocytes. Created by BioRender.com. **(B)** WT mice were treated with low doses of LPS for 3 weeks as depicted in **(A)**. On day 0, day 15 and day 22 bleeding was performed for the determination of IL-6 and TNF-α. Data are shown as mean ± S.D. ****P < 0.0001, one-way ANOVA with the Bonferroni *post hoc* analysis. 5 mice per group (n = 2). **(C)** Heat map representation of cytokine content in serum from mice treated as **(A)** at different time points. Shown is heatmap with colored boxes, representing cytokines amounts ranging from bright blue (lowest) to bright red (highest). Numbers represent cytokine concentrations (pg/ml). **(D)** WT mice were treated as in B and circulating EPCs were determined by flow cytometry. Data are shown as means ± S.D. *P < 0.05, paired samples *t* test. 5 mice per group (n=2). ns, not significant.

To mimic the pathophysiological effects of low-grade inflammatory conditions WT mice were treated with injection of either low doses of LPS (1µg/kg, daily) or PBS as control group for 3 consecutive weeks (**Figure 1A**) and the temporal trend of their circulating EPCs was examined over the treatment period in relation to the concomitant changes in serum cytokines. As expected, 3-week LPS treatment resulted in modest but significant increases in selected proinflammatory cytokines, including IL-6 and TNF-α, compared with the PBS-treated control (**Figure 1B**). Conversely, no significant change occurred in the serum anti-inflammatory cytokines at any time point after initiation of either LPS or PBS treatment (**Figure 1C**). In the same conditions, analysis of systemic EPCs revealed a dramatic decrease in circulating EPCs in mice treated with low doses of LPS (69% decrease from baseline mean value, p=0.039) compared to those treated with the vehicle PBS (**Figure 1D**). These results suggest that although repetitive low-dose LPS induced small cytokine release, it led to a significant decrease in circulating EPC levels.

### Exogenous administration of the metabolite l-kynurenine increases the number of circulating EPCs under homeostatic conditions

IDO1 is induced and activated by various inflammatory stimuli and there is ample evidence that activation of the IDO1 enzyme causes immunosuppression in several chronic inflammatory conditions, e.g., autoimmune diseases, bacterial and viral infections, and in various tumors (35, 36). Tryptophan metabolism by IDO1 produces several metabolites including l-kyn which is characterized by immunoregulatory functions (22) and potential vasoactive properties (37).

**Figure 2:**
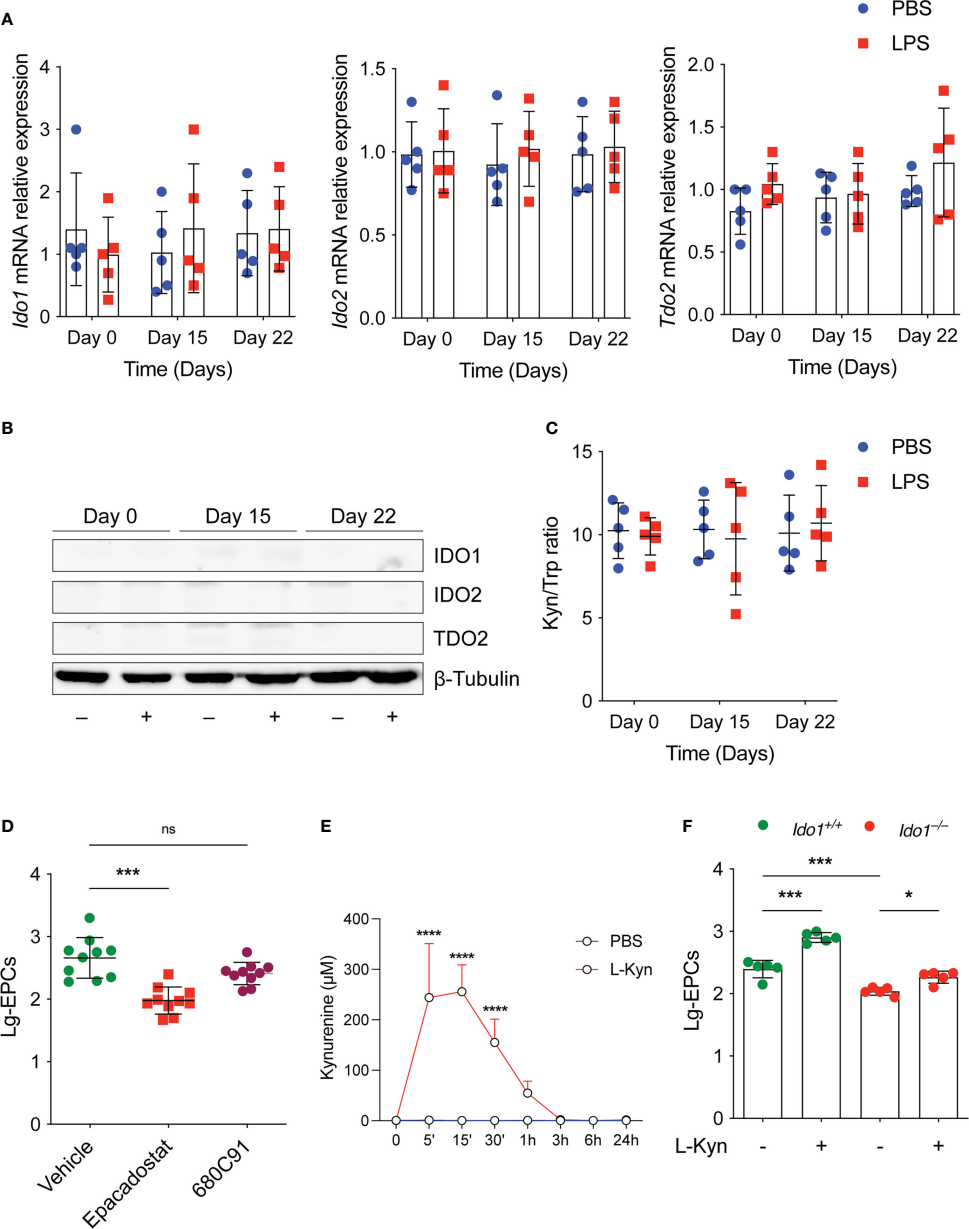
The tryptophan metabolism preserves EPCs numbers *in vivo* under homeostatic conditions. **(A)** *Ido1* and *Ido2* mRNA expression was assessed by RT-PCR on day 0, day 15 and day 22 in splenocytes derived from mice treated either with PBS or LPS. Data are represented as normalized transcript expression in the samples relative to normalized transcript expression in control mice. Data are shown as mean ± S.D. One-way ANOVA with the Bonferroni *post hoc* analysis. 5 mice per group (n=2). **(B)** Western analysis was carried out for IDO1 and IDO2 protein expression in total splenocytes derived from mice in **(A)** β-Tubulin was used ad loading control. One representative experiment (n = 3). **(C)** Serum l-kyn content serum was determined by HPLC in mice treated as in **(A)** Data are shown as mean ± S.D. One-way ANOVA with the Bonferroni *post hoc* analysis. 5 mice per group (n=2). **(D)** The number of circulating EPCs was evaluated in WT mice treated with IDO1 (Epacadostat) or TDO2 (680C91) inhibitors or vehicle as control under homeostatic conditions by flow cytometry. ***P < 0.001, one-way ANOVA followed by Bonferroni multiple comparison test. 10 mice per group (n=2). **(E)** Pharmacokinetic of oral administration of l-kyn. 2 mg of l-kyn were administrated by oral gavage and the l-kyn concentration was monitored in the plasma at indicates time points. Data are shown as mean ± S.D. ****P < 0.0001, two-way ANOVA with the Bonferroni post hoc analysis. 3 mice per group (n = 2). **(F)** The number of circulating EPCs was evaluated in WT and *Ido1^–/–^* mice treated with PBS or l-kyn for 3 weeks by flow cytometry. *P < 0.5, ***P < 0.001, one-way ANOVA followed by Bonferroni multiple comparison test. 5 mice per group (n=2). ns, not significant.

We studied the expression and activity of tryptophan metabolic enzymes including IDO1, IDO2 and TDO2, in experimental-induced low-grade inflammation. Surprisingly, we found that low-dose conditioning with LPS *in vivo* (**Figure 1A**) did not result in neither in increased *Ido1, Ido2* and *Tdo2* transcript (**Figure 2A**), or protein (**Figure 2B**) in the spleens and PBMCs (data not shown) of treated mice, analyzed at different time points. Accordingly, the l-kyn/Trp ratio in plasma of the same mice was not increased by the same LPS conditioning (**Figure 2C**). Thus, although various inflammatory signals, including LPS, have been reported to induce Trp metabolism, these data suggest that there may be a specific threshold at which such induction occurs.

Next, we examined the effects of key tryptophan-metabolizing enzymes such as IDO1 and TDO2 on circulating EPCs under homeostatic conditions using selective enzyme inhibitors for each of the enzymes. To this end, we compared the number of EPCs in WT mice treated with vehicle or with a selective IDO1 or TDO2 inhibitor *in vivo*. We found that homeostatic kynurenine production affected EPCs. Specifically, we found that the number of circulating EPCs was significantly lower in mice treated with the IDO1 inhibitor (**Figure 2D**), and a trend toward decreased EPCs was also observed in mice treated with the TDO2 inhibitor (**Figure 2D**), although this was not statistically significant. Notably, oral administration of l-kyn resulted in significant systemic increase of its concentration *in vivo* (**Figure 2E**). Moreover, multiple weekly treatments with l-kyn to *Ido1^–/–^* mice restored normal circulating EPC numbers as those found in WT mice (**Figure 2F**). Similarly, we showed that the same l-kyn treatment significantly increased circulating EPCs in WT mice (**Figure 2F**).

**Figure 3:**
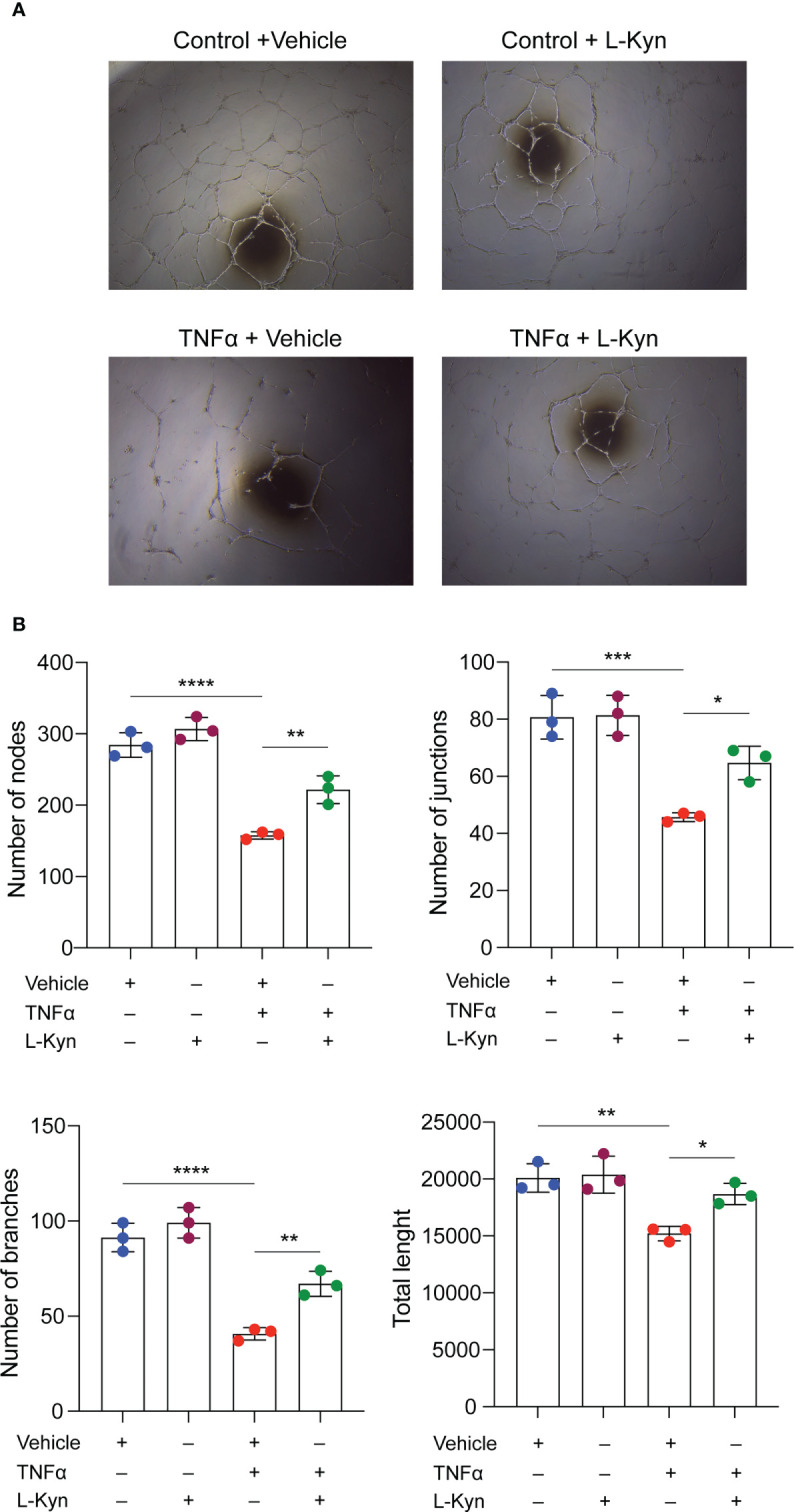
l-kynurenine prevents TNF-α effects on the formation of capillary networks. **(A)** Representative images of tubulogenic assay on BOECs in presence or absence of TNF-α and/or l-kyn. Vehicle was used as control. Scale bar = 500 μm. **(B)** Quantification of number of nodes, branches, junctions and total length of the tubule networks. ****P < 0.0001; ***p < 0.001; **p 0.01; *p < 0.05. Data are expressed as mean ± S.D. and are representative of three independent experiments. ns, not significant.

To further elucidate the mechanism by which l-kyn contributes to the maintenance of higher numbers of EPCs, we tested whether l-kyn also exerts a protective effect on endothelial cells *in vitro* by using BOECs (or ECFCs) for tubulogenic assay. For this purpose, we treated the cells with the proinflammatory cytokine TNF-α as a cytokine induced by chronic stimulation with low dose of LPS. Multiple treatments with this cytokine impaired the formation of capillary networks, whereas the addition of l-kyn to the medium markedly enhanced the formation of tubule networks, as shown in **Figure 3A**. Quantification of the network formed showed an increase in the number of nodes, branches, junctions, and total length of tubules (**Figure 3B**), indicating that l-kyn protected cells from inflammatory injury during tubulogenicity assay.

Overall, these data suggest that Trp metabolism plays a role in homeostatic maintenance of EPC numbers *in vivo*.

### The Trp metabolite l-kyn prevents the decline in circulating EPCs in mice treated with a low-dose of LPS

**Figure 4:**
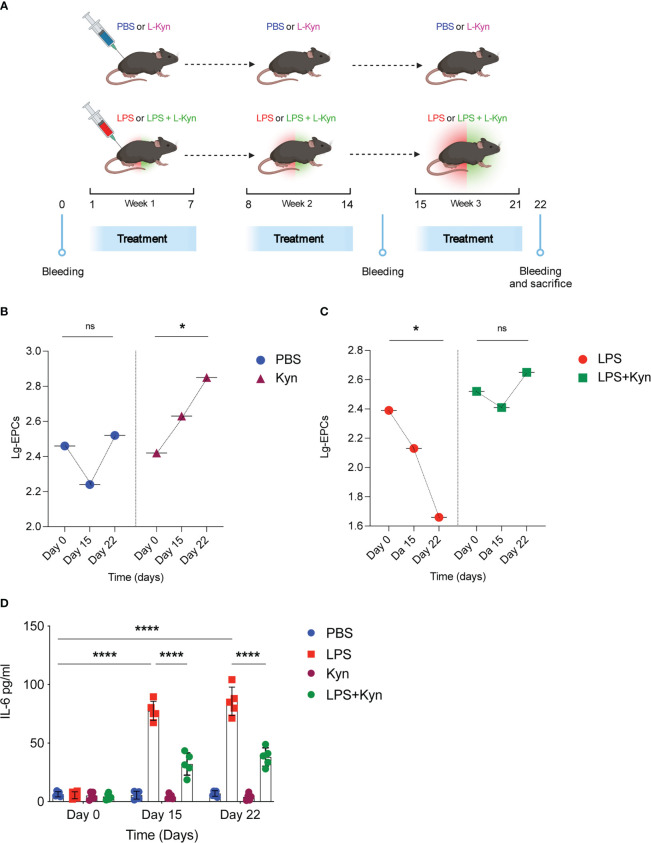
l-kynurenine administration prevents EPC numbers reduction during low-grade inflammation. **(A)** WT mice were treated with low doses of LPS in combination with l-kyn for 3 weeks. On day 0, day 15 and day 22 bleeding was performed for the determination of circulating EPCs and pro-inflammatory cytokines. Created by BioRender.com. **(B, C)** The number of circulating EPCs was evaluated by flow cytometry on day 0, day 15 and day 22 in mice treated with either l-kyn or LPS or combination of both. Data are shown as means ± S.D. *P < 0.05, paired sample t test. 5 mice per group (n=2). **(D)** Serum derived from mice treated as in **(A-C)** was assessed for IL-6 production. Data are shown as means ± S.D. ****P < 0.0001, one-way ANOVA with the Bonferroni *post hoc* analysis. 5 mice per group (n=2). ns, not significant.

To examine the impact of the Trp metabolite l-kyn on circulating EPCs in homeostatic conditions overtime, the effect of oral l-kyn administration on circulating EPCs was compared with that of PBS treatment in WT mice (**Figure 4A**). Accordingly, to the **Figure 2E**, the oral l-kyn administration significantly increases circulating EPCs progressively over the weeks of treatment (18% increase from baseline mean value, p=0,034) as compared to PBS alone (F=3,790, p=0,045, for the interaction between the type of treatment and circulating EPCs) (**Figure 4B**). Next, to experimentally investigate the ability of l-kyn to influence the relationship between circulating EPCs and systemic low-grade inflammation, the temporal trend of circulating EPCs and serum cytokines was compared between WT mice treated with l-kyn and LPS and WT mice treated with LPS alone (**Figure 4A**). While circulating EPCs decreased significantly in LPS-treated WT mice (69% reduction from baseline mean value, p=0,039), they did not significantly change over the treatment period in WT mice receiving the combined treatment with l-kyn and LPS (p=0,466) (F=4,168, p=0,035 for the interaction between the type of treatment and circulating EPCs) (**Figure 4C**). In addition, the temporal trend of serum pro-inflammatory cytokines was compared between the two treatment groups. While a significant decrease of serum IL-6 levels occurred in WT mice receiving the combined treatment with l-kyn and LPS, a significant increase of IL-6 was recorded as compared to baseline in WT mice receiving LPS treatment alone (**Figure 4D**).

Overall, the present data suggest that l-kyn levels might be a potentially protective endogenous metabolite associated with lower EPC decline in low-grade inflammation conditions.

### IDO1 activity influences circulating EPC levels in patients with variable systemic low-grade inflammation

Based on the preclinical results in mice we exploited the potential association between circulating EPC numbers and kyn/Trp levels in patients characterized by variable systemic low-grade inflammation (i.e., subclinical inflammation). The main clinical and biochemical characteristics of the human study population (n=277) according to low *versus* high IDO1 activity [i.e., kyn/Trp <40 (low IDO1 activity) versus kyn/Trp ≥40 (high IDO1 activity)] are summarized in **Table 1**. Patients with low kyn/Trp ratio (n=120) and those with high kyn/Trp ratio (n=157) were balanced in terms of age and sex. Patients with high IDO1 activity had significantly higher BMI, waist circumference, circulating EPCs, and hs-CRP levels as compared with patients with low kyn/Trp ratio.

**Table 1:** Characteristics of the human study population according to low IDO1 activity versus high IDO1 activity (i.e., Kyn/Trp <40 versus Kyn/Trp ≥40).

Patient baseline Characteristics	Kyn/Trp <40 (n=120)	Kyn/Trp ≥40 (n=157)	p
**Age, years**	59 ± 14	62 ± 14	0.056
**Sex, % men**	43	40	0.626
**BMI, kg/m^2^**	27 ± 5	31 ± 10	<0.001
**WC, cm**	90 (85-100)	107 (90-133)	<0.001
**Current smoking, %**	34	25	0.111
**Diabetes, %**	4	10	0.093
**SBP, mmHg**	129 ± 15	132 ± 12	0.103
**DBP, mmHg**	78 ± 9	79 ± 8	0.418
**Total cholesterol, mg/dL**	214 ± 44	201 ± 44	0.027
**LDL cholesterol, mg/dL**	143 ± 40	131 ± 37	0.016
**HDL cholesterol, mg/L**	51 ± 14	51 ± 16	0.503
**Triglycerides, mg/dL**	106 (80-159)	115 (87-149)	0.316
**Glucose, mg/dL**	91 (83-100)	91 (83-99)	0.949
**eGFR, mL/min**	82 (22)	78 (19)	0.269
**EPCs, cell/mL**	123 (75-212)	198 (99-369)	<0.001
**Hs-CRP, mg/L**	1 (0.3-4.1)	1.8 (0.7-4)	0.011

Values are expressed as percentage, mean ± standard deviation or median (interquartile range). BMI, body mass index; DBP, diastolic blood pressure; eGFR, estimated glomerular filtration rate (Cockcroft-Gault equation); EPCs, endothelial progenitor cells; HDL, high-density lipoprotein; hs-CRP, high-sensitivity C-reactive protein; Kyn, kynurenine; LDL, low-density lipoprotein; Lg, logarithmic; SBP, systolic blood pressure; Trp, tryptophan; WC, waist circumference.

**Figure 5:**
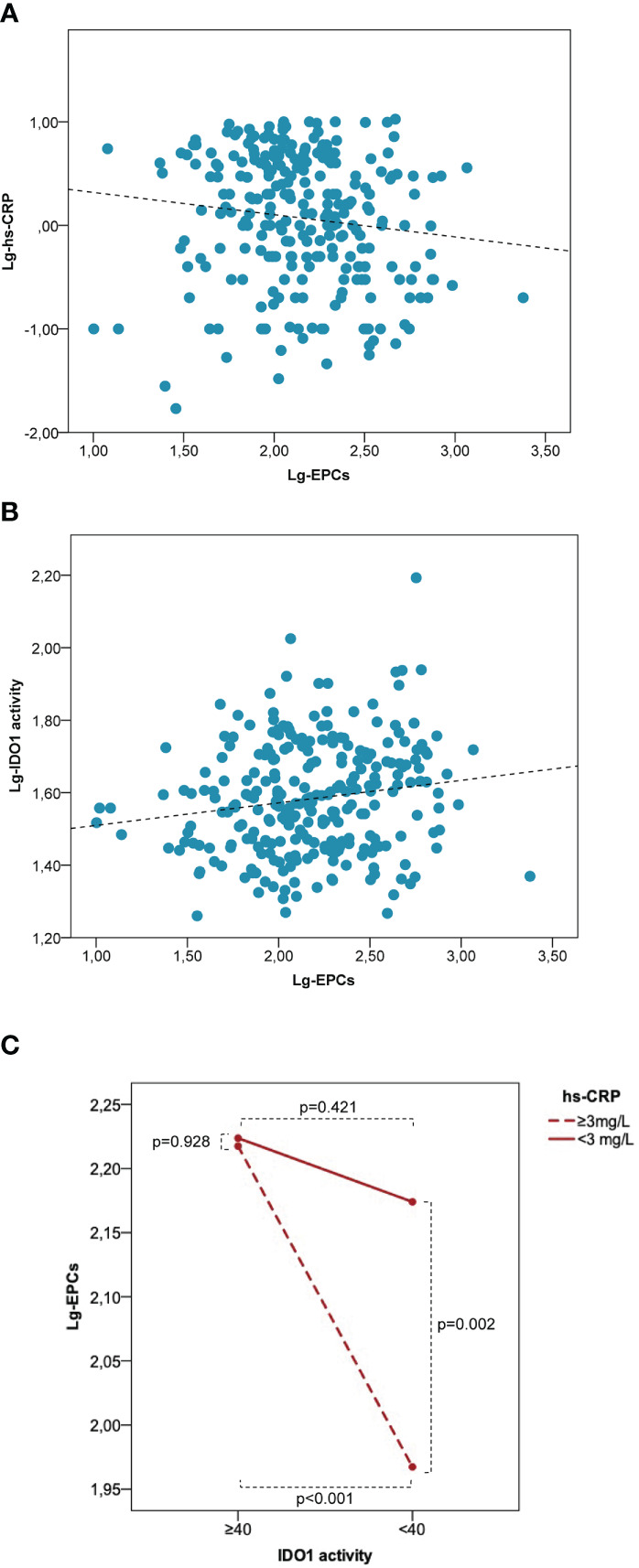
Low IDO1 activity is associated with a decrease of EPC numbers in patients characterized by low-grade inflammation. Correlation between lg-EPCs and lg-hs-CRP **(A)** and between lg-EPCs and lg-IDO1 activity **(B)** in the human study population. **(C)** Interaction between IDO1 activity and hs-CRP in the prediction of circulating EPCs. EPCs, endothelial progenitor cells; hs-CRP, high-sensitivity C-reactive protein; IDO1, indoleamine 2,3-dioxygenase 1; lg, logarithmic. Patients with variable low-grade systemic inflammation (n = 277) were stratified according to low versus high IDO1 activity (i.e., Kyn/Trp <40 versus Kyn/Trp ≥40) and high versus low hs-CRP (i.e., < 3 mg/L versus ≥3 mg/L). High hs-CRP is associated with significantly lower EPC numbers as compared with low hs-CRP at low IDO1 activity but not at high IDO1 activity. P values are from the independent sample *t* test.

In the entire study population, lg-EPCs were significantly associated with age (r=-0.121, p=0.046), BMI (r=0.205, p=0.003), total cholesterol (r=-0.157, p=0.015), lg-hs-CRP (r=-0.125, p=0.044) (**Figure 5A**), and lg-kyn (r=0.158, p=0.008) (**Figure 5B**). Lg-kyn correlated significantly with age (r=0.132, p=0.029), BMI (r=0.286, p<0.001), total cholesterol (r=-0.183, p<0.004), LDL cholesterol (r=-0.226, p=0.001), and lg-hs-CRP (r=0.297, p<0.001). Lg-hs-CRP was significantly associated with BMI (r=0.175, p=0.014) and systolic blood pressure (r=0.214, p=0.003). While a significant inverse association was found between lg-EPCs and lg-hsCRP in patients with low kyn/Trp ratio (r=-0.226, p=0.005), no significant association emerged between lg-EPCs and lg-hsCRP in patients with high kyn/Trp ratio (r=-0.006, p=0.954) (**Supplementary Figure 3**). A significant kyn/Trp ratio (low versus high) × hs-CRP (low versus high) interaction was found in the prediction of circulating EPCs (p_interaction_=0.038). In patients with high hs-CRP, circulating EPC levels were significantly lower in presence of low kyn/Trp ratio as compared to high kyn/Trp ratio (p<0.001). Conversely, in patients with low hs-CRP no significant difference emerged in circulating EPCs according to kyn/Trp ratio (p=0.421). Also, in patients with low kyn/Trp ratio circulating EPCs were significant lower in the high hs-CRP subgroup as compared to the low hs-CRP subgroup (p=0,002). Instead, in patients with high kyn/Trp ratio circulating EPC levels were comparable between the high and low hs-CRP subgroups (p=0.928) (**Figure 5C**). Thus, the trend of hsCRP and EPCs according to high versus low Kyn/Trp ratio which is shown in **Table 1** risks is not fully representative of the complex relationship between hsCRP, EPCs and Kyn/Trp ratio under disease conditions underlined by low-grade inflammation.

**Figure 6:**
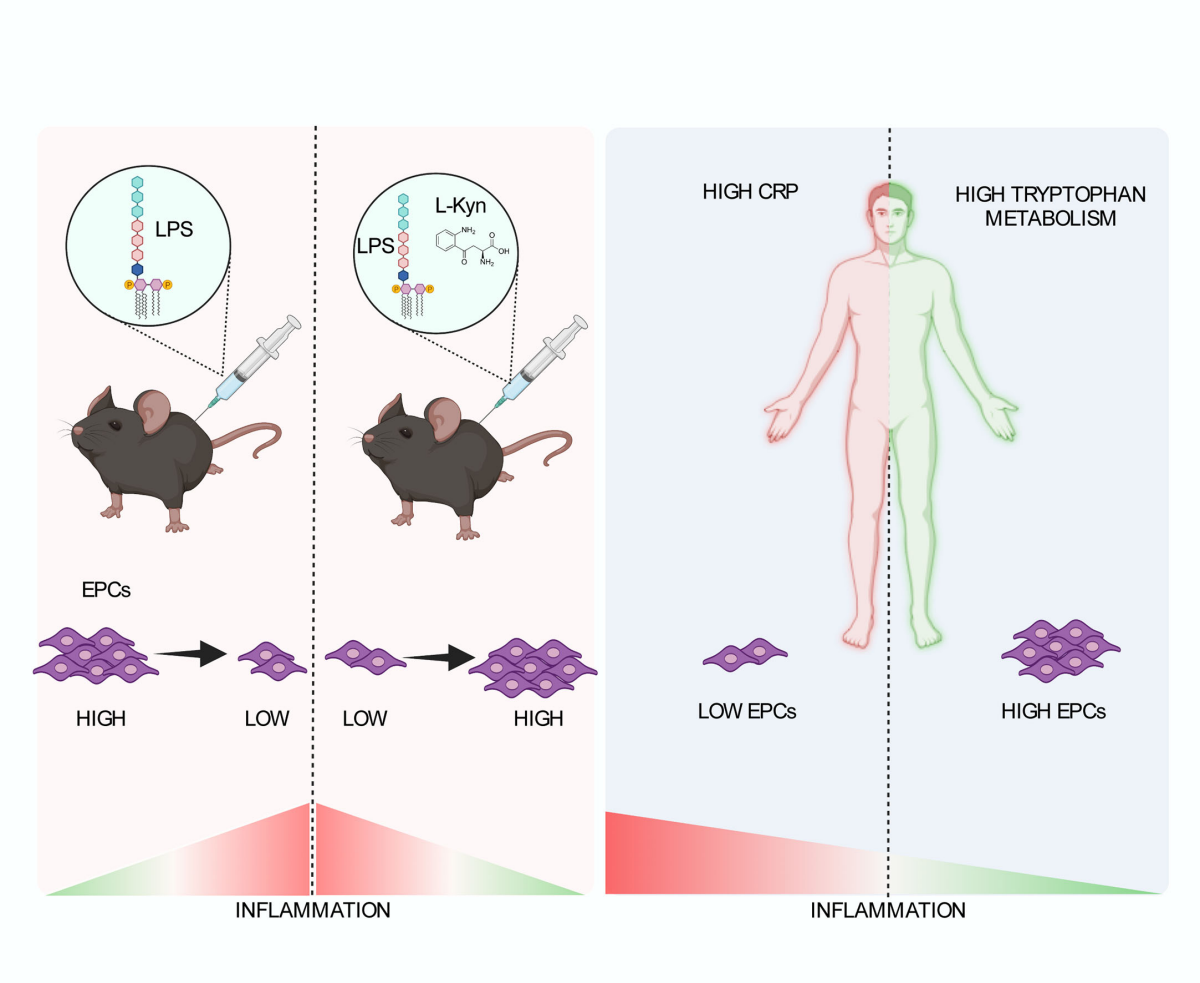
Schematic representation of the role of tryptophan metabolism in the low-grade inflammation leading to the maintenance of EPC numbers. In a preclinical model of systemic low-grade inflammation, repetitive low-dose LPS administration reduces circulating EPC levels. Moreover, l-kyn supplementation limit circulating EPC loss (left). In human beings (right), high hs-CRP is associated with significantly lower EPC counts as compared to low hs-CRP in the presence of low Kyn/Trp ratio but not high Kyn/Trp ratio, while low Kyn/Trp ratio is associated with lower circulating EPCs as compared to high Kyn/Trp ratio in the presence of high hs-CRP but not low hs-CRP. Created by BioRender.com.

## Discussion

In this study, a complementary approach (animal model and human model) was used to investigate the influence of IDO1 activity on circulating EPCs in the presence of systemic low-grade inflammation. A first result to discuss is that both in the animal model and in the human model circulating EPC counts were found to be negatively influenced by systemic low-grade inflammation. Indeed, in WT mice repetitive low doses of LPS induced a significant decline of circulating EPCs in parallel with a significant increase of serum IL-6 and TNF-α levels. In addition, in the human study an inverse association emerged between hs-CRP and circulating EPCs. Overall, these findings confirm those from a number of previous studies showing a detrimental impact of low dose endotoxemia/chronic subclinical inflammation on serum EPC availability (38–42). Noteworthy, in the animal model the induction of systemic low-grade inflammation did not influence IDO1, IDO2 or TDO2 expression and activity. Although this may apparently seem in contrast with consolidated evidence showing that IDO1 can be induced by inflammatory stimuli, it is in line with previous reports showing that LPS-mediated IDO1 induction is dose-dependent (43). Thus, a lack of IDO1 or other tryptophan metabolizing enzymes, such as TDO2 upregulation could be expected in low dose endotoxemia. Another result worth of discussion is that both in the animal study and in the human study kyn/Trp ratio was found to positively influence serum EPC availability, either in homeostatic conditions or in the presence of systemic low-grade inflammation. Moreover, higher circulating EPC levels were found in WT mice as compared to *Ido1^–/–^* mice or in those treated with the selective IDO1 inhibitor. In addition, l-kyn administration induced a significant increase of circulating EPC counts in WT mice. Also, a positive association emerged between Trp catabolism and circulating EPCs in the human study population. Similarly, in LPS-treated WT mice the Trp metabolite l-kyn prevented circulating EPC decline by inducing a significant decrease of serum IL-6 and TNF-α levels. Interestingly, l-kyn was able to prevent the negative impact of TNF-α on capillary network formation, favoring a significant improvement of the tubule network formation *in vitro*. Although the mechanism by which this effect may be activated remains to be investigated, the reduction in the negative effects of TNF-α on BOEC cells may be related to activation of AhR, which is one of the known targets of L-kyn (24) and also a regulator of the effects and production of TNF-α (44, 45). Moreover, in the human study a significant interaction emerged between IDO1 activity and hs-CRP in the prediction of circulating EPCs (i.e., high hs-CRP was associated with significantly lower EPC counts as compared to low hs-CRP in the presence of low kyn/Trp ratio but not high kyn/Trp ratio, while low kyn/Trp ratio was associated with lower circulating EPCs as compared to high kyn/Trp ratio in the presence of high hs-CRP but not low hs-CRP). All these findings are original and unprecedent. Indeed, to our knowledge, no previous studies have ever assessed the relationship between Trp metabolism and circulating EPCs nor the influence of IDO1 or other Trp metabolizing enzymes on the relationship between systemic low-grade inflammation and circulating EPCs. Overall, although a comprehensive mechanistic explanation of the positive relationship between Trp metabolism and circulating EPC levels either in homeostatic conditions or in the presence of systemic low-grade inflammation has not been provided in this study, it may be speculated that the activation of the l-kyn pathway by preferentially IDO1 activity may somehow favour EPC survival or EPC mobilization from bone marrow potentially either by an intrinsic or extrinsic effect of the Trp degrading enzyme or their metabolites on the EPCs. Also, it may be speculated that IDO1 activity in selected cells or tissues mediates protective effects on circulating EPCs may rely on a possible IDO1-mediated anti-inflammatory activity at least in the presence of systemic low-grade inflammation. In line with the first speculation, it has been reported that the activation of the l-kyn pathway by IDO1 is crucial for the proliferation and survival of human pluripotent stem cells (hiPSC), which are cellular precursors of EPCs, whereas the knockdown of IDO1 by small interfering RNA has been shown to inhibit hiPSC cell growth (46). Also, in IDO1 expressing cells the inhibition of IDO1 activity has been associated with a reduced expression of CXCR4 (42, 43), a chemokine receptor with a key role in the regulation of hematopoietic stem cell mobilization (44); this suggests that IDO1 activity may potentially have an impact in the mobilization of EPCs from bone marrow by regulating the CRCR4 pathway. In line with the second speculation, there is a large body of evidence showing that IDO1 downregulates the inflammatory response in different chronic inflammatory conditions, including chronic subclinical inflammation (21, 23, 25, 47, 48). Thus, for instance, dendritic cells expressing IDO1, and exosomes derived from dendritic cells overexpressing IDO1 have been shown to display immunosuppressive and anti-inflammatory effects in collagen-induced arthritis (49) and in models of multiple sclerosis (36). Also, the overactivation of the IDO1 axis has been associated with reduced inflammation and atherosclerotic burden in hyperlipidemic mice (50), whereas inhibition of IDO1 activity has been linked with increased vascular inflammation and acceleration of atherosclerosis in *Apoe^–/–^* mice (51). However, it cannot be neglected that a possible pro-inflammatory role of IDO1 activity in chronic inflammatory conditions is suggested by some lines of evidence. Thus, for instance, in *Ldlr^–/–^* mice IDO1 activity has been reported to suppress the expression of the anti-inflammatory cytokine IL-10, thereby favouring atherosclerosis progression (52). Also, IDO1 depletion has been shown to induce an anti-inflammatory response in macrophages from mice with chronic viral myocarditis (53). Hence, explanations alternative to IDO1-mediated suppression of inflammation should be considered in order to unfold the mechanisms underlying the positive relationship between IDO1 activity-mediated preservation of circulating EPC counts in presence of systemic low-grade inflammation.

Nonetheless, despite all these speculative considerations being plausible and attractive, future experimental studies are warranted to characterize the molecular pathways underlying the positive impact of l-kyn and IDO1 in selected tissue on circulating EPCs. Particularly, it should be elucidated as to whether IDO1 activity-mediated positive impact on circulating EPC availability may rely or not on the activation of AhR pathway, which has a crucial role in kyn-mediated biological effects. From a translational perspective, the results of the present study may support the hypothesis that the Trp metabolite l-kyn, might favour endothelial reparative and regenerative mechanisms in chronic subclinical inflammation by increasing circulating EPC availability. Therefore, l-kyn administration might represent a possible therapeutic approach against endothelial damage in this condition. To date, no preclinical/clinical studies have attempted to explore the role of kynurenine mimetics to this aim, therefore, results of future studies are awaited especially in patients with reduced levels of l-kyn. Some limitations of this study should be discussed, as well. First, in the animal model systemic low-grade inflammation was induced with a single scheme of low-dose LPS. This was chosen in order to demonstrate the possible independent role of systemic low-grade inflammation in the modulation of circulating EPC levels by avoiding the possible confounding effect of concomitant IDO1 or other enzyme upregulation at least in lymphoid organs. However, as to whether different schemes of LPS administration may induce a significant increase of IDO1 activity, thereby producing a different EPC response, needs to be clarified. Second, a comprehensive mechanistic explanation of the association between l-kyn supplementation and EPC levels either in homeostatic conditions or in systemic low-grade inflammation was not formally provided. Therefore, only speculative considerations on putative underlying mechanisms can be drawn although we showed for the first time a positive impact of l-kyn on endothelial tubulogenesis. Fourth, the oral supplementation with l-kyn was performed in order to investigate its gross effect on circulating EPC bioavailability rather than to define a possible dose-response curve. Therefore, caution is needed when evaluating quantitatively the effect of l-kyn supplementation on circulating EPC availability. Moreover, only a trend in EPC increase was detected by the addition of l-kyn or by increasing IDO1 expression on *in vitro* bone marrow cultures. Thus, these preliminary data suggest that the effects of l-kyn in regulating EPC numbers may involve a more complex system and potential cell extrinsic and intrinsic effects on EPC progenitor cells (**Figure 6**). Further experiments are needed to address this question and will be performed in future studies. In conclusion, this study confirms that systemic low-grade inflammation reduces EPC levels and shows for the first time that IDO1 activity and l-kyn supplementation may improve EPC availability by limiting circulating EPC loss induced by systemic low-grade inflammation. Accordingly, it would be of interest to evaluate the stimulation of the kynurenine pathway as a possible therapeutic approach to counteract endothelial damage in chronic inflammatory conditions.

## Data availability statement

The original contributions presented in the study are included in the article/**Supplementary Material**. Further inquiries can be directed to the corresponding authors.

## Ethics statement

The studies involving human participants were reviewed and approved by Ethical Committee of the University of Perugia. The patients/participants provided their written informed consent to participate in this study. The animal study was reviewed and approved by Italian ministry of health.

## Author contributions

Conceptualization, MP, MG and FF. Methodology, MG, FF. Formal analysis, VB and MM. Investigation, MG, VB, GS, GM, GMe, DR, BP, LF, AC, AF and FB. Resources, MM, VB, MP, FG, FFi, EC, CC, PP and DF. Data curation, MM and VB. Writing—original draft preparation, FF. Writing—review and editing, FF. Visualization, VB and MG. Supervision, FF. Project administration, MG and FF. Funding acquisition, FF. All authors contributed to the article and approved the submitted version.
