# Supplementary material for: A tryptophan metabolite prevents depletion of circulating endothelial progenitor cells in systemic low-grade inflammation
Source: Front Immunol. 2023 Apr 4;14:964660. doi: 10.3389/fimmu.2023.964660 (PMC10110845; doi:10.3389/fimmu.2023.964660)
Supplement: Supplementary file 1 [file DataSheet_1.pdf]

SUPPLEMENTARY INFORMATION

A tryptophan metabolite prevents depletion of circulating endothelial progenitor cells in systemic low-grade inflammation

Massimo R. Mannarino[1,3], Vanessa Bianconi[1,3‡], Giulia Scalisi[1], Luca Franceschini[1], Giorgia Manni[1], Alessia Cucci[2], Francesco Bagaglia[1], Giulia Mencarelli[1], Francesco Giglioni[1], Doriana Ricciuti[1], Filippo Figorilli[1], Benedetta Pieroni[1], Elena Cosentini[1], Eleonora Padiglioni[1], Cecilia Colangelo[1], Dietmar Fuchs[2], Paolo Puccetti[1], Antonia Follenzi[2], Matteo Pirro[1], Marco Gargaro[1,4‡], Francesca Fallarino[1,4‡]

‡ Vanessa Bianconi, Ph.D., MD (vanessa.bianconi@unipg.it)
‡ Marco Gargaro, Ph.D. (marco.gargaro@unipg.it)
‡ Francesca Fallarino, Ph.D. (francesca.fallarino@unipg.it)

---

[1] Department of Medicine and Surgery, University of Perugia, Perugia, Italy;
[2] Department of Health Sciences, School of Medicine, University of Piemonte Orientale, 28100 Novara, Italy;
[3] Division of Biological Chemistry, Biocenter, Innsbruck Medical University, Austria;
[4] These authors contributed equally to this work;
[5] These authors share senior authorship on this paper;

This Supplementary File includes:

Supplementary Figures 1 to 3 with Legends

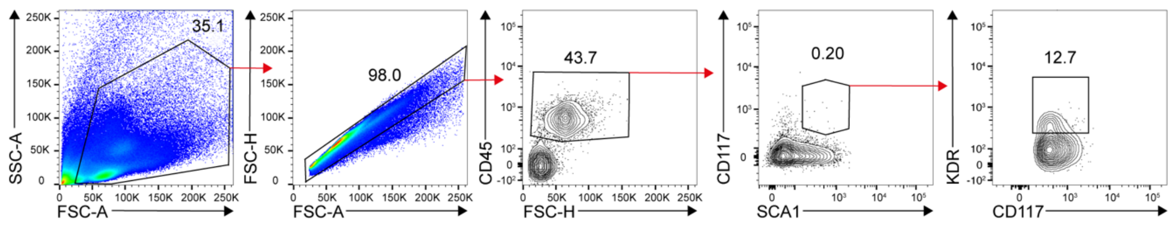

Supplementary 1. Gating strategy of EPCs from the blood of mice. Cells were isolated from the blood of WT mice. EPCs were gated as $CD45^{+}CD117^{+}SCA1^{+}KDR^{+}$. Numbers indicate the percentage of cells in the indicated gates.

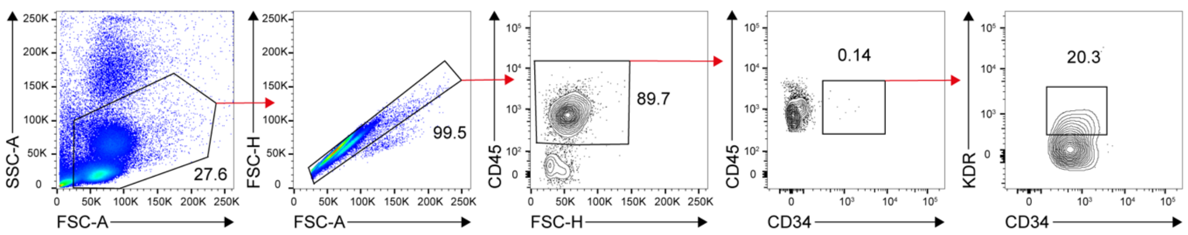

Supplementary 2. Gating strategy of EPCs from human blood. PBMCs were isolated from human blood. EPCs were gated as $CD45^{+}CD34^{+}KDR^{+}$. Numbers indicate the percentage of cells in the indicated gates.

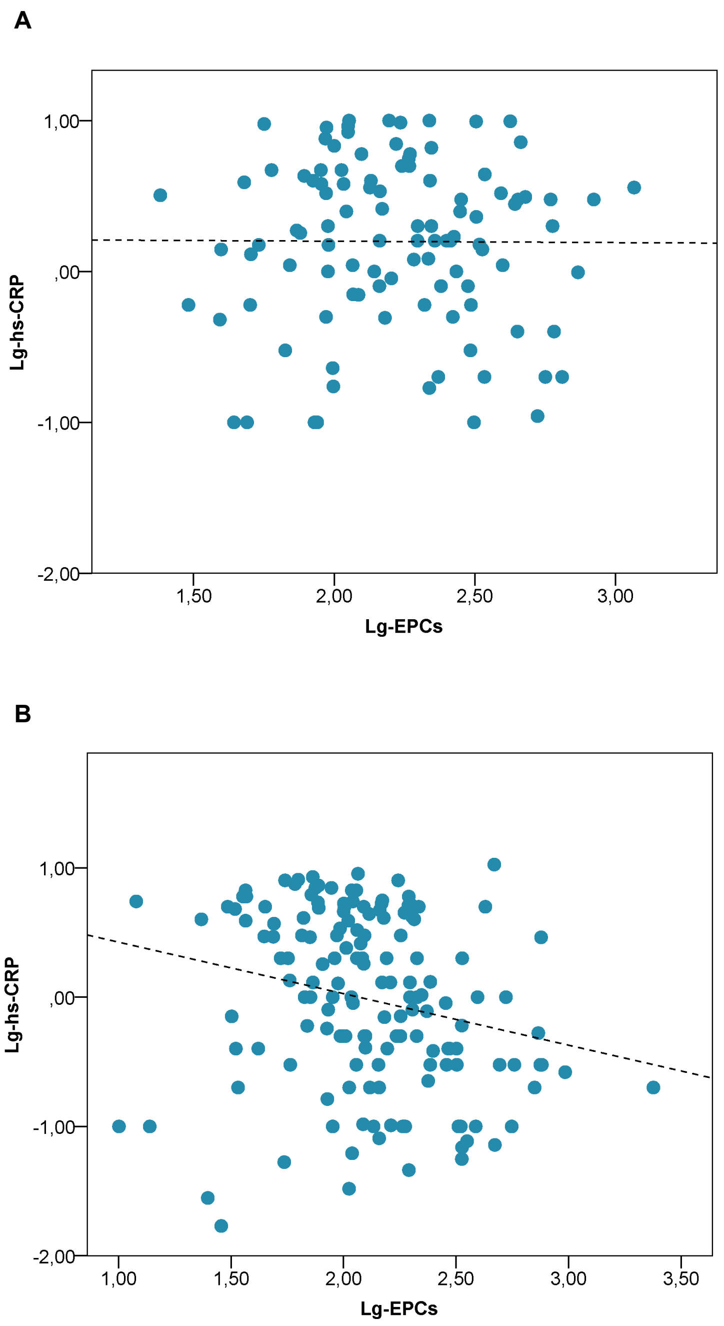

Supplementary Figure 3. EPC numbers are inversely associated with hs-CRP in patients with low Kyn/Trp but not in patients with high Kyn/Trp. Correlation between lg-EPCs and lg-hs-CRP in patients with high Kyn/Trp (A) and correlation between lg-EPCs and lg-hs-CRP in patients with low Kyn/Trp (B). EPCs, endothelial progenitor cells; hs-CRP, high-sensitivity C-reactive protein; Kyn, kynurenine; lg, logarithmic; Trp, tryptophan. Patients with variable low-grade systemic inflammation ($n = 277$) were stratified according to low versus high Kyn/Trp (i.e., Kyn/Trp <40 versus Kyn/Trp$\geq$ 40). EPC numbers are inversely associated with hs-CRP in patients with low Kyn/Trp ($r=-0.226$, $p=0.005$) but not in patients with high Kyn/Trp ($r=-0.006$, $p=0.954$). P values are from the Pearson's correlation.
